# Chronomodulated Administration of Chemotherapy in Advanced Colorectal Cancer: A Systematic Review and Meta-Analysis

**DOI:** 10.7759/cureus.36522

**Published:** 2023-03-22

**Authors:** Ahmed Nassar, Amir Abdelhamid, George Ramsay, Mohamed Bekheit

**Affiliations:** 1 The Health Services Research Unit, University of Aberdeen, Aberdeen, GBR; 2 Aberdeen Royal Infirmary, National Health Service (NHS) Grampian, Aberdeen, GBR; 3 Dr Gray’s Hospital, National Health Service (NHS) Grampian, Aberdeen, GBR; 4 HPB Centre, Elite Integrated Centres of Excellence, Alexandria, EGY

**Keywords:** colorectal cancer, chronomodulation, circadian rhythm adjusted chemotherapy, chemotherapy for advanced colorectal cancer, chronomodulated chemotherapy

## Abstract

In this systematic review, the efficacy and safety of chronomodulated chemotherapy, defined as the delivery of chemotherapy timed according to the human circadian rhythm, were assessed and compared to continuous infusion chemotherapy for patients with advanced colorectal cancer.

Electronic English-language studies published until October 2020 were searched. Randomised controlled trials (RCTs) comparing chronomodulated chemotherapy with non-chronomodulated (conventional) chemotherapy for the management of advanced colorectal cancer were included. The main outcomes were the objective response rate (ORR) and system-specific and overall toxicity related to chemotherapy. Electronic databases including Ovid Medline, Ovid Embase, Cochrane Central Register of Controlled Trials and the Cochrane Database of Systematic Review were searched.

In total, seven RCTs including 1,137 patients were analysed. Males represented 684 (60%) of the study population. The median age was 60.5 (range = 47.2-64) years. There was no significant difference between chronomodulated and conventional chemotherapy in ORR (risk ratio (RR) = 1.15; 95% confidence interval (CI) = 0.87-1.53). Similarly, there was no significant difference in gastrointestinal toxicity under the random effect model (RR = 1.02; 95% CI = 0.68-1.51). No significant difference was found regarding neurological and skin toxicities (RR = 0.64, 95% CI = 0.32-1.270 and RR = 2.11, 95% CI = 0.33-13.32, respectively). However, patients who received chronomodulated chemotherapy had less haematological toxicity (RR = 0.36, 95% CI = 0.27-0.48).

In conclusion, there was no overall difference in ORR or haematologic toxicity between chronomodulated and non-chronomodulated chemotherapy used for patients with advanced colorectal cancer. Chronomodulated chemotherapy can be considered in patients at high risk of haematological toxicities.

## Introduction and background

Circadian rhythm is based on two main mechanisms. The first mechanism is a central system which acts as a coordinator and includes suprachiasmatic nuclei located in the hypothalamus and considered the main circadian pacemaker [1], and the second mechanism is a molecular *clock* which is present in most cells in the brain and peripheral tissue and consists of multiple feedback loops produced by transcriptional and post-transcriptional process triggered by genes responsible for the expression of specific proteins in a rhythmic manner [1,2]. Effects on cell division cycle-related changes such as apoptosis and cell repair pave the way to investigate developing cancer chemotherapeutic regimens [1,3].

The circadian rhythm plays a role in several biological processes. At least 15 specific genes are believed to be related to circadian rhythm participating in controlling cell proliferation, DNA replication, apoptosis, angiogenesis, metabolism, and drug detoxification [4,5]. There is an observed 24-hour change in the activities of several enzymes involved in the catabolism of different chemotherapeutic factors or the anabolism of their cytotoxic forms [6-8]. Hence, circadian rhythm can be related to the efficacy of cancer treatment under what is known as chronotherapy, which is an approach that can potentially improve the tolerability and efficacy of chemotherapy [5].

Chronomodulation of chemotherapy is based on utilising the circadian rhythm to increase the efficacy of anti-neoplastic agents [9]. It has been found that some isoforms of heat shock protein (HSP) 90, which mediate cell-cycle progression, show the circadian pattern of expression, which may explain the circadian rhythm-dependent efficacy of some anti-cancer agents [10,11]. Moreover, the toxic effects of endotoxins [12] and the anti-cancer agent, cyclophosphamide, show dependency on the time of the day [13,14].

Over the last two decades, several experimental and clinical studies have shown a favourable association between adjusting timing and dosing of chemotherapeutic agents according to circadian rhythm and response in cancer patients [3]. L-alanosine is an amino acid analogue derived from the bacterium Streptomyces alanosinicus and shows anti-metabolic and potential anti-neoplastic activity [15]. It has shown a selective in vitro anti-neoplastic activity against methylthioadenosine phosphorylase (MTAP)-deficient tumours [1,16] such as leukaemias, brain tumours, non-small-cell lung cancers, breast cancers, melanomas, pancreatic cancers, and sarcomas [17-21]. However, bone marrow suppression and mucositis are common causes of dose limitation and discontinuation [1,22]. One study in mice has proven a three-fold decrease in mortality with circadian rhythm-adjusted doses of L-alanosine, confirming a potentially strong role of circadian rhythm [1]. Chronomodulation of chemotherapy is yet to become standard practice in other disease settings, and it remains unclear if such an approach may influence outcomes.

Colorectal cancer is the fourth most common cancer in the United Kingdom, accounting for 11% of all new cancer cases [23], and it is the second most common fatal cancer after lung cancer in western countries [6,24]. Metastases are detected in 25-30% of patients at the time of diagnosis and develop during the disease course in a further 25% of patients [6]. Metastases are responsible for 90% of deaths from colorectal cancer [25], the majority of which are seen in the liver [26]. In these advanced cases, chemotherapy is indicated for the control of the systemic disease, which may not be controlled by surgery only [4,6,27].

As a result of the rates of advanced presentation and metastatic disease, chemotherapy regimens are commonplace in colorectal cancer practice. However, the optimal mechanism by which such therapy is delivered remains unclear. Fluorouracil (5-FU) and leucovorin (folinic acid) (LV) are included in most chemotherapy regimens for colorectal cancer and result in an objective response (i.e. decrease in tumour size by 50% or more) in 20-25% of patients and up to 50% if combined with other agents such as oxaliplatin with a dose-related response [5,28,29]. This indicates that if chemotherapy is tolerated well, the patient may benefit from the full therapeutic dose [29]. However, between 31% and 34% of patients experience severe toxicity from 5-FU [30]. Myelosuppression resulting in severe neutropenia and anaemia is the main toxicity of 5-FU [31]. Gastrointestinal toxicity resulting in diarrhoea and mucositis can occur but is less frequent [31]. Likewise, oxaliplatin is associated with anaemia which can be severe if combined with 5-FU [32]. Preoperative anaemia in colorectal cancer is associated with poor disease progression and postoperative recovery [33]. Additionally, oxaliplatin is associated with neurotoxicity and fatigue [34].

Chronomodulation of chemotherapy is defined as the delivery of chemotherapy with respect to the circadian rhythm in which different doses of chemotherapy are delivered according to the time of the day [24,29,35]. In this approach, circadian rhythm-related changes can be utilised to improve the tolerance and efficacy of chemotherapy [25,36]. There are, however, controversies regarding the tolerability and efficacy of a chronomodulated chemotherapy regimen compared to the conventional (non-chronomodulated) regimen despite the presence of randomised controlled trials (RCTs) comparing these two approaches of chemotherapy for advanced colorectal cancer [25].

Despite studies showing a potential beneficial role of chronomodulation as previously mentioned, its effect on objective response rate (ORR) and different body system toxicities are yet to be proven. In addition, other factors that may affect the results of chronomodulation still need to be explored. Hence, a recent synthesis of the currently published literature in this field is yet to be undertaken. In this review, the efficacy and safety of chronomodulated chemotherapy were assessed and compared to continuous infusion chemotherapy for patients with advanced colorectal cancer.

This article was previously posted to the medRxiv preprint server on December 11, 2022.

## Review

Methods

Study Design

This review was prepared in line with the Preferred Reporting Items for Systematic reviews and Meta-Analyses (PRISMA) statement [37] and was registered with PROSPERO (University of York) before the study selection process (registration number: CRD42020227313).

Inclusion Criteria

Electronic English-language studies published until October 2020 were searched. RCTs comparing chronomodulated chemotherapy with conventional chemotherapy for the management of advanced colorectal cancer were included. The main outcomes were the objective response rate (ORR) and system-specific and overall toxicity related to chemotherapy.

Exclusion Criteria

Observational studies, reviews, and non-RCTs were excluded. A study was excluded if included patients did not have colorectal cancer.

Search Strategy

Electronic databases including Ovid Medline, Ovid Embase, Cochrane Central Register of Controlled Trials (CENTRAL), and the Cochrane Database of Systematic Review (CDSR) were searched. The search was conducted by a senior information specialist from the library department of the Royal College of Surgeons of England and was executed on 27 October 2020.

Study Question

In patients with advanced colorectal cancer, what is the effect of chronomodulated chemotherapy compared to conventional chemotherapy on ORR and chemotherapy-related toxicities?

The Patient Intervention Control Outcome (PICO) framework [38] was used to guide the search (Table 1). The full electronic search strategy is shown in Table 2.

**Table 1:** PICO framework.

PICO	Description	Search terms
Population	Patients diagnosed with colorectal cancer and/or colorectal liver metastasis cancer undergoing chemotherapy	Gastrointestinal neoplasms/or liver neoplasms/or carcinoma, hepatocellular/or biliary tract neoplasms/or bile duct neoplasms/or cholangiocarcinoma/digestive system cancer/or biliary tract tumour/or bile duct carcinoma/or liver tumour/or hepatobiliary system cancer/or liver cell carcinoma/or gallbladder cancer/colon cancer/rectal cancer/
Intervention	Chronomodulated chemotherapy	Chronomodulated chemotherapy, chronotherapy, chronomodulated chemotherapy, chronomodulation
Control	Non-chronomodulated chemotherapy/standard chemotherapy/conventional chemotherapy	Drug therapy/or antineoplastic agents/chemotherapy/chronomodulated chemotherapy/chronotherapy
Outcome	Objective response rate (ORR), toxicity, associated symptoms during chemotherapy (e.g. vomiting, nausea, headache, etc.)	No search terms were used to find more results

**Table 2:** Search in Ovid MEDLINE database.

Database:	Ovid MEDLINE(R) ALL <1946 to October 26, 2020>	Results per line	Number of results
Date of search	27/10/2020		
1	Gastrointestinal Neoplasms/ or Liver Neoplasms/ or Carcinoma, Hepatocellular/ or Biliary Tract Neoplasms/ or Bile Duct Neoplasms/ or Cholangiocarcinoma/	186,092	54
2	(gastrointestinal or gastro-intestinal or “gastro intestinal” or “GI” or hepatobiliary or “HPB” or ?esophag* or pancrea* or stomach or bile or biliary or gallbladder or colon or rectum or rectal or anus or anal or liver or “small intestin*”).ti,ab,kw,kf.	1,931,611	
3	Neoplasms/ or Carcinoma/ or (cancer* or neoplas* or tumo?r* or malignan* or carcinoma* or mesothelioma*).ti,ab,kw,kf.	3,477,984	
4	2 and 3	573,741	
5	1 or 4	658,435	
6	Drug Therapy/ or Antineoplastic Agents/ or (chemotherap* or chemo-therap* or “drug* therap*”).ti,ab,kw,kf.	686,691	
7	5 and 6	90,563	
8	Chronotherapy/ or (chronomodulat* or chrono-modulat* or “chrono modulat*” or chronotherap* or chrono-therap* or “chrono therap*”).ti,ab,kw,kf.	1,820	
9	7 and 8	88	
10	Limit 9 to human	75	
11	Limit 10 to the English language	54	
12	Limit 11 to last 30 years	54	
13	Remove duplicates from 12	54	

Two independent blinded reviewers performed the abstract screening. Any conflicts were resolved by a third reviewer to produce the final list of studies eligible for full-text review. Full-text review and data extraction from individual studies were performed by two researchers, with another researcher confirming the adequacy and accuracy of the extracted data. Data included each study’s details, demographic data, details of chemotherapy regimen, disease characteristics, previous treatment (chemotherapy, radiotherapy, and surgery), ORR, and specific toxicities in both treatment arms. The follow-up period and withdrawals from each study were also noted. The revised Cochrane risk-of-bias tool for RCTs (ROB2) Tool was used to assess the risk of bias from all included RCTs [39].

Definitions

The control group underwent conventional (non-chronomodulated) chemotherapy and was referred to as the control group (group A) while the treatment group underwent chronomodulated chemotherapy and was referred to as the treatment group (group B).

The ORR is the assessment of the tumour burden after a given treatment and was measured according to the World Health Organization (WHO) criteria for disease response [40].

The term toxicity refers to toxicity secondary to chemotherapy involving one or more of the gastrointestinal, haematological, neurological, and dermatological systems. Toxicity was graded according to National Cancer Institute-Common Toxicity Criteria [41] (Table 3).

**Table 3:** Grading of toxicity.

Grade of toxicity	Explanation
Grade 1	Mild; asymptomatic or mild symptoms; clinical or diagnostic observations only; intervention not indicated
Grade 2	Moderate; minimal, local, or non-invasive intervention indicated
Grade 3	Severe or medically significant but not immediately life-threatening; hospitalization or prolongation of hospitalization indicated
Grade 4	Life-threatening consequences; urgent intervention indicated

Chemotherapeutic agents utilized in the included studies included 5-FU, LV (folinic acid), CPT-11, FUDR, and I-OHP: oxalatoplatinum. As different regimens were included in the studies this review relied on, these chemotherapy regimens are defined in Table 4.

**Table 4:** Definitions of chemotherapy regimens. 5-FU: fluorouracil; LV: leucovorin (folic acid); CPT-11: irinotecan; FUDR: floxuridine; I-OHP: oxalatoplatinum

Regimen name	Chemotherapeutic agents included
Regimen 1	5-FU, LV, and oxaliplatin
Regimen 2	Intra-arterial 5-FU and oxaliplatin
Regimen 3	CPT-11, 5-FU, and LV
Regimen 4	Venous 5-FU and arterial FUDR
Regimen 5	5-FU, I-OHP, and LV

Data Handling

Only grade 3 and 4 toxicities were analysed as they can be a cause of interrupting the chemotherapy course or reducing the dose due to non-tolerability.

Statistical Analysis

Count, percentages, and ratios were used to represent categorical variables, and median (range) was used to represent continuous data, as stated in each individual study. Outcomes, such as ORR and toxicity, were represented by risk ratios (RRs) and 95% confidence intervals (CIs).

This meta-analysis was conducted using RevMan (Review Manager) software version 5.4 (Cochrane collaboration, United Kingdom). Analysis was done for different types of toxicities. I^2^ and Tau^2^ values were used to assess heterogeneity. If the I^2^ value was >50%, significant heterogeneity was considered, and Mantel-Haenszel (M-H) random effect model was employed [42]. Meta-regression was performed using the Comprehensive Meta-analysis software version 3. A significant difference was considered if the p-value was less than 0.05. Funnel plots were produced to visualize the risk of publication bias across studies, and significant asymmetry was an indication of publication bias.

Ethical Review

This was a meta-analysis of data already published in RCTs, and thus ethical review was not required.

Results

Studies Characteristics

**Figure 1:**
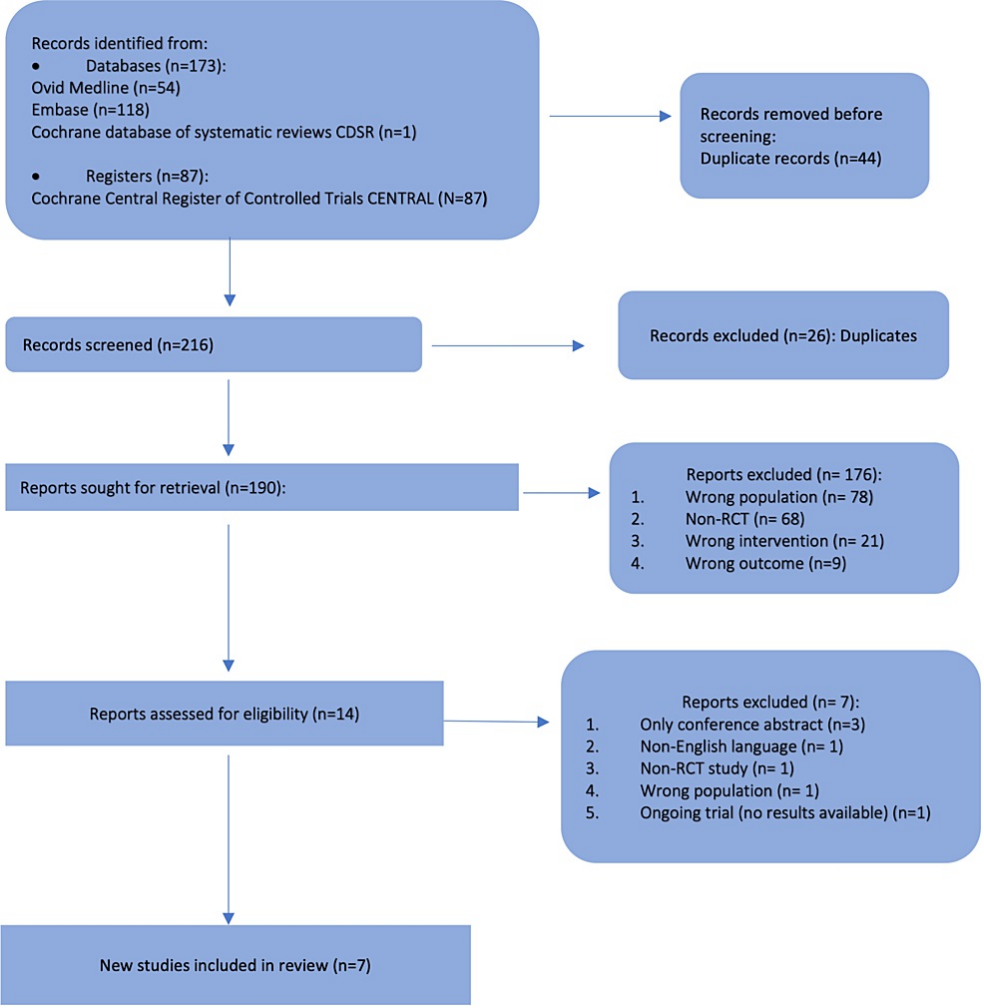
Preferred Reporting Items for Systematic Reviews and Meta-Analyses flow diagram.

Out of the 260 studies identified in the search, 70 were duplicates. After screening and checking eligibility, seven RCTs were included in the quantitative analysis. Figure 1 displays the selection stratification (in PRISMA format). All included RCTs were of parallel randomised design except Levi et al.’s 1997 [29] study, which was designed as a cluster-randomised trial. All trials included patients with advanced colorectal cancer with or without metastasis who needed chemotherapy. Five trials were multicentre studies [29,35,43-45]. The main study characteristics, including inclusion and exclusion criteria, are summarised in Table 5. There were differences in the inclusion and exclusion criteria of eligible patients across studies. In one study by Ramanathan et al., four treatment arms were compared [43]. All of them received the same regimen (Regimen 1) but at different timings except in arm 1 where LV was not included. Hence, arm 1 (n = 23) was excluded from the meta-analysis. Arms 2 and 3 were considered as group A (conventional chemotherapy) (n = 81) and arm 4 as group B (chronomodulated chemotherapy) (n = 25).

**Table 5:** Study characteristics. Group A: control group (conventional chemotherapy); Group B: intervention group (chronomodulated chemotherapy); PFS: progression-free survival; OS: overall survival; NS: not specified; CNS: central nervous system; AST: aspartate transaminase; ORR: objective response rate

Study	Setting	Target population	Inclusion criteria	Exclusion criteria	Comparison	Patient withdrawals	Conclusion
Ramanathan et al. 2008 [41]	Multicentre16: sites in the United States and Canada	Patients with locally advanced or metastatic adenocarcinoma of the colon or rectum	Age >18, Karnofsky performance >60%, fertile males or females had to use contraceptives	Concurrent, active non-colorectal primary, serum creatinine level >1.25 (ULN); total bilirubin level >2 times the ULN or serum AST level >2 times the ULN, unless liver metastases were present; or absolute neutrophil count (ANC) <1.5 × 10^9^/L or a platelet count <100 × 10^9^/L	Response, toxicity/response rate, PFS	Not specified	No advantage to using chronomodulated chemotherapy in terms of toxicity and efficacy
Huang et al. 2006 [44]	Not identified	Patients with colorectal liver metastasis with previously removed primary confirmed adenocarcinoma	Only liver metastasis; no previous liver metastasis-directed treatment; the primary was removed	NS	Toxicity (WHO grades)/response rate	Not specified	Decreased toxicity in the chronomodulation group but the same response rate
Giacchetti et al. 2006 [42]	Multicentre, phase III, 36 institutions, 10 countries	Patients with metastatic colorectal cancer	WHO performance status less or equal to 2; adenocarcinoma; age from 18 to 76 years; adequate hematologic, renal, and hepatic functions; measurable metastatic lesions (largest diameter more than 20 mm); no brain metastases; and no prior chemotherapy or radiotherapy for metastatic disease	NS	The two-year survival rate, PFS, ORR, quality of life	Two from A and four from B	Chronomodulation produced improved survival in men
Garufi et al. 2006 [24]	Not identified	Patients with colorectal adenocarcinoma with unresectable metastasis	Aged between 18 and 80 years; life expectancy >3 months; WHO performance status of 0–2; measurable equal or more than 2 cm or evaluable disease and prior therapy	Serious medical illness, CNS metastases, or a previous history of other malignancies (except for excised cervical or basal skin/squamous cell carcinoma)	Primary outcome: ORR; secondary outcome: dose-intensity, toxicity, PFS, and survival	Three from A and four from B had a non-measurable disease	Less toxicity and PFS in the intervention arm
Focan et al. 1999 [43]	Two centres in France	Patients with unresectable liver metastasis from colorectal cancer	Age younger than 76 years, Karnofsky score more than 60. measurable unresectable liver metastasis	More than two extrahepatic nodules, previous hepatic-directed therapy, serious medical condition	Maximum tolerated dose/Toxicity ORR	Seven from A and two from B before the sixth course	Chronomodulation allowed for increased doses and tolerability
Lévi et al. 1997 [29]	Multicentre, nine institutions in three countries	Patients with measurable metastases from colorectal cancer	Patients with measurable metastases from colorectal cancer	NS	Response rate, survival	Six from A	Clinical relevance of chronotherapy and call for its integration into the early stages of anticancer drug development
Lévi et al. 1994 [35]	Multicentre: seven centres in France, Italy, and Belgium	Patients with metastatic colorectal cancer	Measurable metastasis, life expectancy of more than a month	Surgically resectable metastasis, cerebral metastasis, age above 75, WHO performance status more than 2, previous chemo/radiotherapy for metastasis	Response rate, PFS, and toxicity	Not specified	Chronotherapy improved the response rate

Risk of Bias and Quality Assessment

One trial was of high risk of bias [45], four trials had some concerns [35,43,44,46], and two were of low risk of bias according to the ROB2 assessment tool [24,29]. Further details are presented in Table 6.

**Table 6:** Assessment of risk of bias (ROB2 tool). ROB2: Risk of Bias 2

Study	Randomisation process	Bias due to deviations from the intended interventions (effect of assignment to intervention)	Bias due to deviations from the intended interventions (effect of adhering to intervention)	Risk of bias due to missing outcome data	Risk of bias in the measurement of the outcome	Risk of bias in the selection of the reported result	Overall risk of bias
Ramanathan et al. 2008 [43]	Some concerns	Some concerns	Low	Low	Low	Low	Some concerns
Huang et al. 2006 [46]	Low	Some concerns	Low	Low	Low	Low	Some concerns
Giacchetti et al. 2006 [44]	Low	Low	Some concerns	Low	Low	Low	Some concerns
Garufi et al. 2006 [24]	Low	Low	Low	Low	Low	Low	Low risk
Focan et al. 1999 [45]	Low	Some concerns	Some concerns	Some concerns	Low	Low	High risk
Lévi et al. 1997 [29]	Low	Low	Low	Low	Low	Low	Low risk
Lévi et al. 1994 [35]	Low	Some concerns	Low	Low	Low	Low	Some concerns

The number of patient withdrawals from each trial was noted as 18 patients from the conventional treatment group and 10 patients from the chronomodulated group from four studies [24,29,44,45]. The number of withdrawals was not specified in the other three studies [35,43,46].

**Figure 2:**
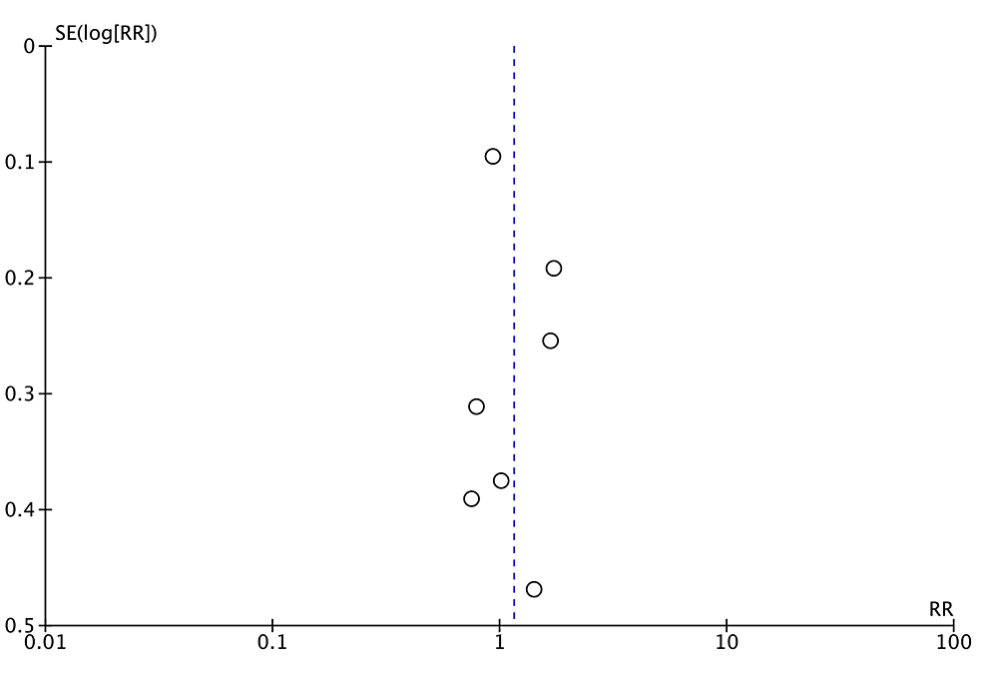
Funnel plot (objective response rate).

**Figure 3:**
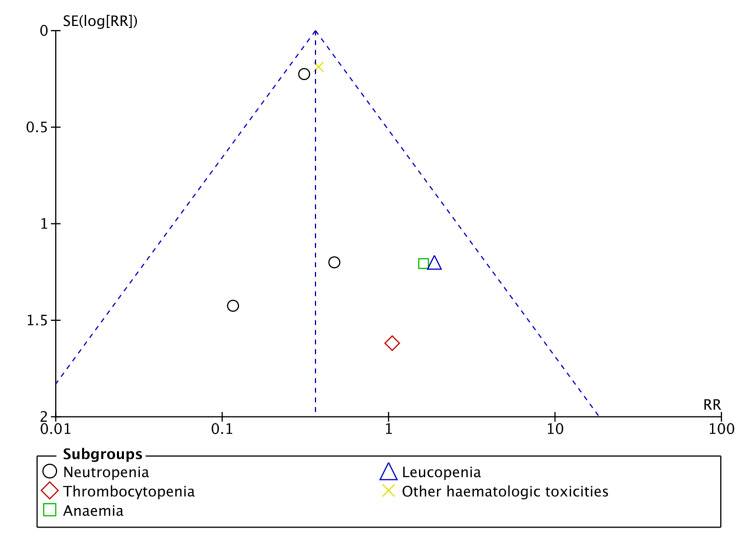
Funnel plot (haematological toxicity).

**Figure 4:**
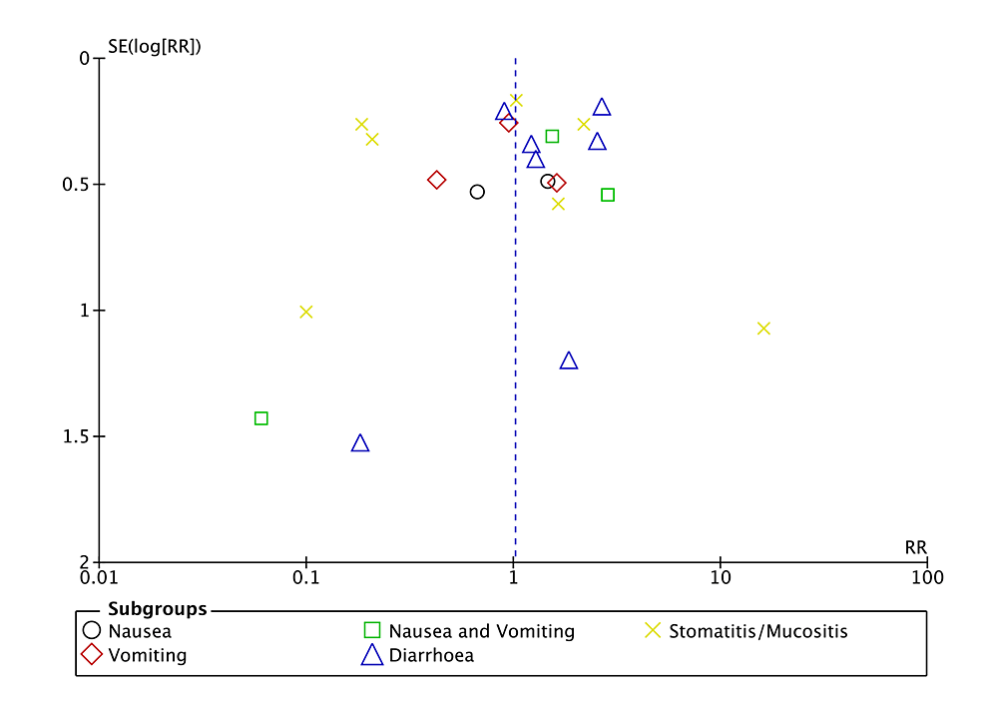
Funnel plot (gastrointestinal toxicity).

There was no significant risk of publication bias regarding ORR and haematological toxicity (Figures 2, 3). However, there was a significant risk of bias in gastrointestinal toxicity (Figure 4). A sufficient number of studies were not available to assess the risk of publication bias in other types of toxicities.

Patient Characteristics

In total, from the seven RCTs, 1,137 patients were included. Males represented 684 (60%) of the study population. The median age was 60.5 (range = 47.2-64) years. The majority of patients (90%) were of 0 and 1 WHO performance status in two studies [24,44]. Karnofsky performance score was more than 60% in two studies [43,45]. There was no reported significant difference in patient characteristics between the two treatment groups in each individual study. A detailed description of patient characteristics is presented in Table 7.

**Table 7:** Patient characteristics. Group A: conventional (non-chronomodulated) chemotherapy; group B: chronomodulated chemotherapy

Study	Sample size	Group A	Group B	Age, median (range)		Gender (M:F)		Performance status (WHO)	
				Group A	Group B	Group A	Group B	Group A	Group B
Ramanathan et al. 2008 [43]	129	104	25	Two arms: 60 (27–78) and 55 (25–75)	61 (43:81)	59:22, 72.8% males	18:7, 72% males	0:55 (67.9%) 1:25 (30.8%) 2:1 (1%)	0:15 (60%) 1:10 (40%) 2:0
Huang et al. 2006 [46]	42	20	22	47.2	53.4	8:12, 40% males	11:11, 50% males	Not specified	Not specified
Giacchetti et al. 2006 [44]	564	282	282	62 (31.8–76)	62 (22.3–76)	170:112, 61% males	168:114, 59.5% males	0:139 (49%) 1:116 (41%) 2:27 (10%)	0:134 (47%) 1:115 (41%) 2:33 (12%)
Garufi et al. 2006 [24]	68	33	35	62 (35–77)	61 (28–77)	19:14, 57.5% males	22:13, 62.8% males	0:22 (67%) 1:9 (27%) 2:2 (6%)	0:22 (63%) 1:12 (34%) 2:1 (3%)
Focan et al. 1997 [45]	56	27	29	58 (34–75)	64 (44–75)	17:10, 63% males	18:11, 62% males	0:11 (41%) 1:14 (52%) 2:2 (7%)	0:12 (41%) 1:16 (56%) 2:1 (3%)
Lévi et al. 1997 [29]	186	93	93	61 (29–75)	61 (22–75)	60:33, 64.5% males	52:41, 56% males	0:50 (54%) 1:34 (37%) 2-3:9 (10%)	0:49 (53%) 1:30 (32%) 2-3:14 (15%)
Lévi et al. 1994 [35]	92	47	45	60 (34–73)	60 (31–73)	27:20, 57.4% males	20:25, 44.4% males	0:14 (30%) 1:27 (57%) 2:6 (13%)	0:19 (42%) 1:23 (51%) 2: 3 (7%)

Disease Characteristics

The colon was the primary site in 842 (74%) patients compared to the rectum which was the primary site in 295 (26%) patients. In four studies, 418 (46%) of patients had metastasis in two or more sites [24,29,35,44]. In the same studies, the liver was involved in 757 (83%) patients and the lung was involved in 322 (35%) patients [24,29,35,44]. In the study by Focan et al., 45 (80%) patients had isolated liver involvement [45]. The study by Huang et al. included patients with only liver metastasis, and 28 (66.7%) patients had both lobes involved [46]. In total, 455 (73%) patients were staged initially as Duke’s stage D (synchronous metastasis) in two studies [44,45]. Moreover, 162 (16.7%) patients had previous chemotherapy and 74 (7.6%) patients had previous radiotherapy in five studies [24,29,35,44,45]. Focan et al. reported a significant discrepancy between both treatment groups regarding patients who had previous therapy (chemotherapy, radiotherapy, or combined), wherein six (22%) patients had prior therapy in group A compared with 15 (52%) patients in group B [45] (Table 8).

**Table 8:** Disease characteristics. Group A: conventional (non-chronomodulated) chemotherapy; Group B: chronomodulated chemotherapy

Study	Primary site - colon:rectum (ratio)		Metastasis		Previous chemotherapy		Previous radiotherapy		Previous surgery	
	Group A	Group B	Group A	Group B	Group A	Group B	Group A	Group B	Group A	Group B
Ramanathan et al. 2008 [43]	76:28 (2.7)	16:9 (1.7)	Not specified		Not specified		Not specified		Not specified	
Huang et al. 2006 [46]	14:6 (2.3)	16:6 (2.6)	Liver metastasis >2 cm: 17 (85%). Both lobes: 12	Liver metastasis >2 cm: 18 (81.8%). Both lobes: 16	Not specified		Not specified		Not specified	
Giacchetti et al. 2006 [44]	213:69 (3)	217:65 (3.3)	Three or more metastasis: 48 (17%)	Three or more metastasis: 38 (13%)	48 (17%)	54 (19%)	18 (6%)	26 (9%)	Surgery for metastasis: 14 (5%)	Surgery for metastasis: 14 (5%)
Garufi et al. 2006 [24]	28:5 (5.6)	28:7 (4)	Two or more: 10 (30%)	Two or more: 13 (37%)	9 (27%)	7 (20%)	2 (6%)	3 (9%)	Not specified	
Focan et al. 1999 [45]	18:9 (2)	23:6 (3.8)	Isolated liver metastasis: 22 (81.4%)	Isolated liver metastasis: 23 (79.3%)	6 (22%)	10 (34%)	0 (0%)	3 (10.3%)	27 (100%)	29 (100%)
Lévi et al. 1997 [29]	66:27 (2.4)	63:30 (2.1)	Less than three: 85 (91%)	Less than three: 85 (91%)	11 (12%)	10 (11%)	7 (8%)	6 (6%)	Surgery for metastasis: 7 (8%)	22 (24%)
Lévi et al. 1994 [35]	30:17 (1.76)	34:11 (3.1)	Two or more: 20 (42.5%)	Two or more: 23 (51.1%)	2 (4.2%)	5 (11.1%)	4 (8.5%)	5 (11.1%)	Not specified	

Chemotherapy Regimen

All chemotherapeutic agents were delivered through a programmable pump via intravenous (IV) access except for two trials (Focan et al. and Huang et al.) [45,46], wherein access to the hepatic artery was established before commencing the first course. The chemotherapeutic agents used were similar in the studies by Ramanathan et al. and Giacchetti et al. [43,44]. 5-FU, LV, and oxaliplatin were used in both studies (Regimen 1). In the study by Garufi et al., chronomodulated 5-FU and LV were administered in both treatment groups but chronomodulated irinotecan was given in the intervention group only (Regimen 3) [24]. Chronomodulation of a specific agent was arranged to ensure peak flow at either 04:00 or 16:00 if a second chronomodulated agent was administered (Table 9).

**Table 9:** Chemotherapy regimens.

Study	Regimen	Duration	Method of delivery	Chronomodulation strategy
Ramanathan et al. 2008 [43]	Regimen 1: 5-FU, LV, oxaliplatin	Up to 24 weeks or disease progression	IV	Only 5-FU was chronomodulated with a five-hour infusion with a peak at 04:00. Oxaliplatin was administered immediately before 5-FU over six hours on day one every three weeks
Huang et al. 2006 [46]	Regimen 2: Arterial 5-FU and oxaliplatin	Each patient arranged to receive two courses, followed by further chemotherapy or radio-ablation depending on the response	Seldinger technique to cannulate the hepatic artery properly if metastasis on both lobes. Right or left hepatic artery cannulation if confined to one lobe. Gastroduodenal artery embolisation if necessary. Porth Cath System (PCS) subcutaneously over the inguinal region	5-FU infused from 22:00 to 10:00 with peak flow at 04:00. Oxaliplatin infused between 10:00 and 22:00 with peak flow at 16:00
Giacchetti et al. 2006 [44]	Regimen 1: 5-FU, LV, oxaliplatin	Four days for a chronomodulated and two days for a non-chronomodulated course. Courses were repeated every 14 days	IV	5-FU and LV from 22:15 to 09:45 with a peak at 04:00, oxaliplatin from 10:15 to 21:45 with a peak at 16:00
Garufi et al. 2006 [24]	Regimen 3: Irinotecan (CPT-11), 5-FU, LV (both groups received chronomodulated 5-FU and LV)	A five-day course every two weeks, continued until progression, unacceptable toxicity, or patient refusal	IV	CPT-11 was given as a six-hour sinusoidal infusion from 02:00 to 08:00 with peak flow at 05:00 in 250 mL of 5% dextrose
Focan et al. 1999 [45]	Regimen 4: Venous 5-FU, arterial FUDR	A five-day course followed by 16 days treatment-free intervals for at least six courses	All patients had staging laparotomy, cholecystectomy, and gastroduodenal artery ligation at the time of surgical placement of the catheter into the hepatic artery	A peak at 04:00 for 5-FU and 16:00 for FUDR
Lévi et al. 1997 [29]	Regimen 1: 5-FU, LV, oxaliplatin	Each five-day course was repeated after a 16-day interval	IV	Peak flow at 04:00 for 5-FU and 16:00 for oxaliplatin
Lévi et al. 1994 [35]	Regimen 5: 5-FU, I-OHP, LV	Each five-day course was repeated after a 16-day interval for at least six cycles and then offered surgery in case of response	IV	A peak at 04:00 for 5-FU and LV. A peak at 16:00 for I-OHP

Objective Response Rate

In the study by Ramanathan et al., ORR was measured at weeks six, 12, and 18 after the start of treatment and at day 28 post-treatment [43]. Two trials (Giacchetti et al. and Huang et al.) assessed ORR after two and four courses, respectively [44,46]. Three trials measured ORR after every third course [29,35,45].

**Figure 5:**
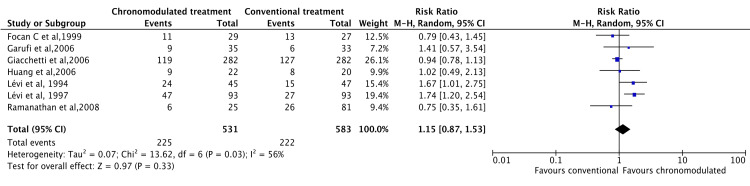
Objective response rate: chronomodulated vs. conventional chemotherapy. Focan et al., 1999 [45]; Garufi et al., 2006 [24]; Giacchetti et al., 2006 [44]; Huang et al., 2006 [46]; Lévi et al., 1994 [35]; Lévi et al., 1997 [29]; Ramanathan et al., 2008 [43].

A meta-analysis was conducted using data from all studies. Under the random effect model, there was no significant difference between chronomodulated and conventional chemotherapy regarding ORR (RR = 1.15, 95% CI = 0.87-1.53) (Figure 5).

Toxicity

**Figure 6:**
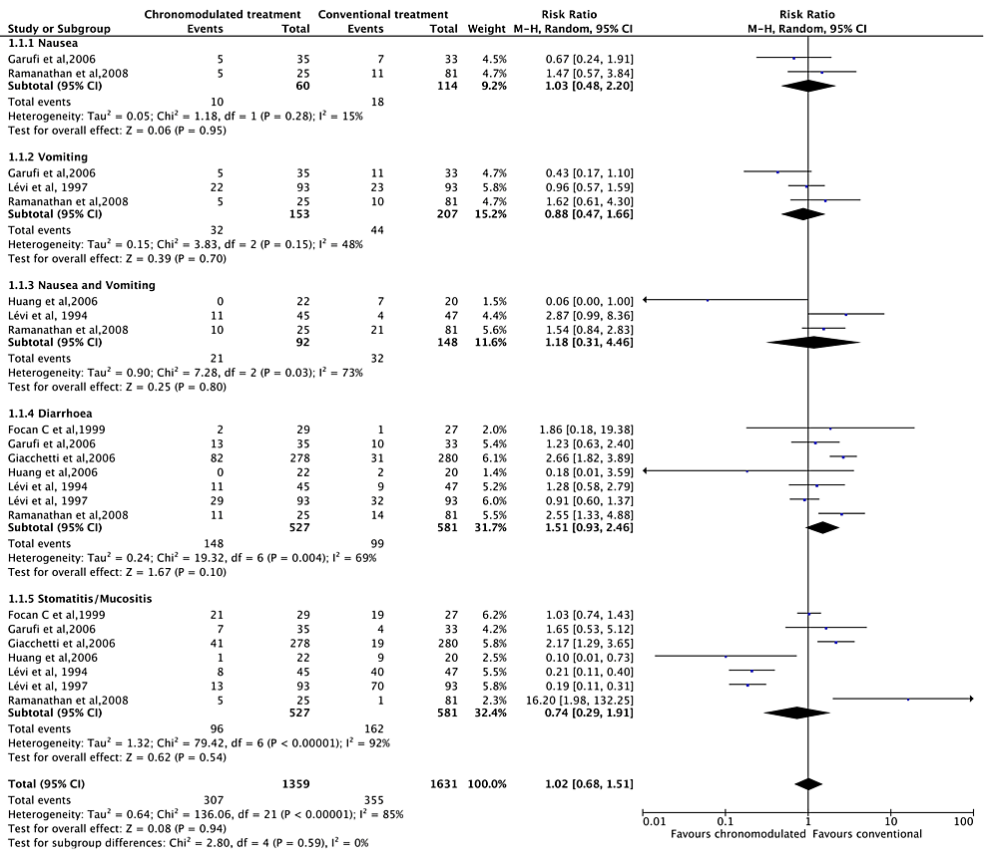
Grade 3 and 4 gastrointestinal toxicities: Chronomodulated vs. conventional chemotherapy. Focan et al., 1999 [45]; Garufi et al., 2006 [24]; Giacchetti et al., 2006 [44]; Huang et al., 2006 [46]; Lévi et al., 1994 [35]; Lévi et al., 1997 [29]; Ramanathan et al., 2008 [43].

Grades 3 and 4 toxicity were assessed. Toxicity was measured for four main systems, including gastrointestinal, haematological, neurological, and skin. There was no significant difference in gastrointestinal toxicity under the random effect model (RR = 1.02, 95% CI =0.681.51) (Figure 6).

**Figure 7:**
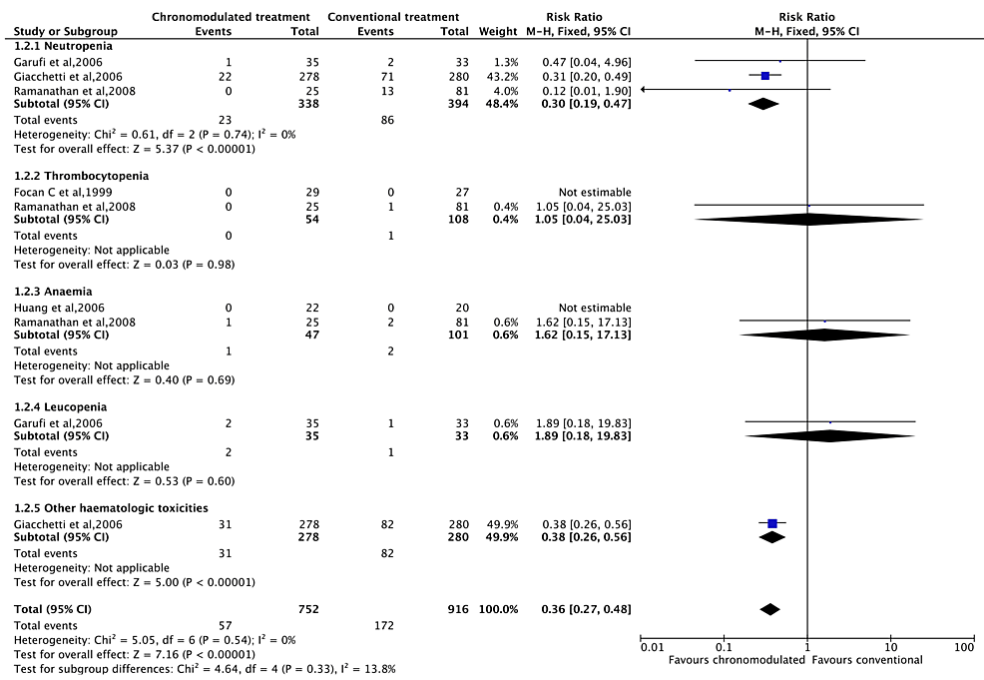
Grade 3 and 4 haematological Toxicities: chronomodulated vs. conventional chemotherapy. Focan et al., 1999 [45]; Garufi et al., 2006 [24]; Giacchetti et al., 2006 [44]; Huang et al., 2006 [46]; Ramanathan et al., 2008 [43].

**Figure 8:**
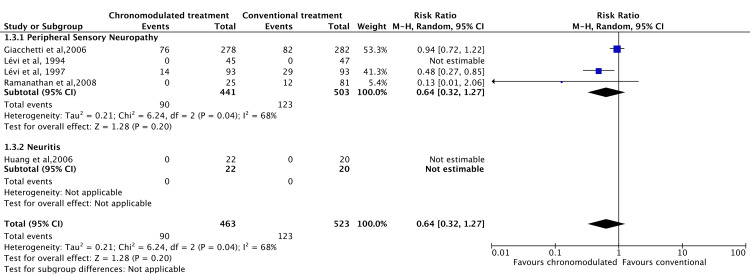
Grade 3 and 4 neurological toxicities: chronomodulated vs. conventional chemotherapy. Giacchetti et al., 2006 [44]; Huang et al., 2006 [46]; Lévi et al., 1994 [35]; Lévi et al., 1997 [29]; Ramanathan et al., 2008 [43].

**Figure 9:**
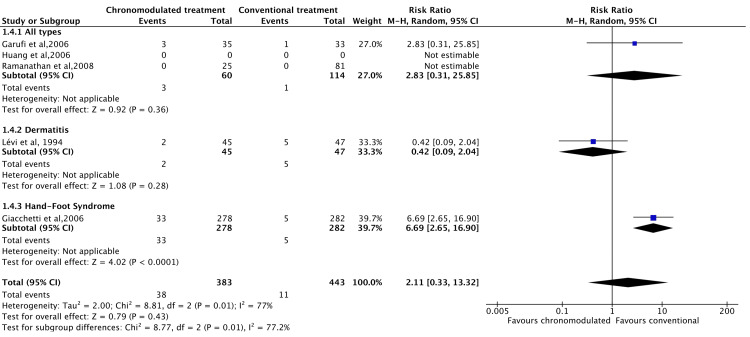
Grade 3 and 4 skin toxicities: chronomodulated vs. conventional chemotherapy. Garufi et al., 2006 [24]; Giacchetti et al., 2006 [44]; Huang et al., 2006 [46]; Lévi et al., 1994 [35]; Ramanathan et al., 2008 [43].

However, the chronomodulated arm had a 63% less chance of developing haematological toxicity (RR = 0.36, 95% CI = 0.27-0.48) (Figure 7). Patients who received chronomodulated chemotherapy had similar neurological toxicities compared to conventional treatment (RR = 0.64, 95% CI = 0.32-1.27) (Figure 8). Similarly, there was no significant difference between both groups regarding skin toxicities (RR = 2.11, 95% CI = 0.33-13.32) (Figure 9).

**Figure 10:**
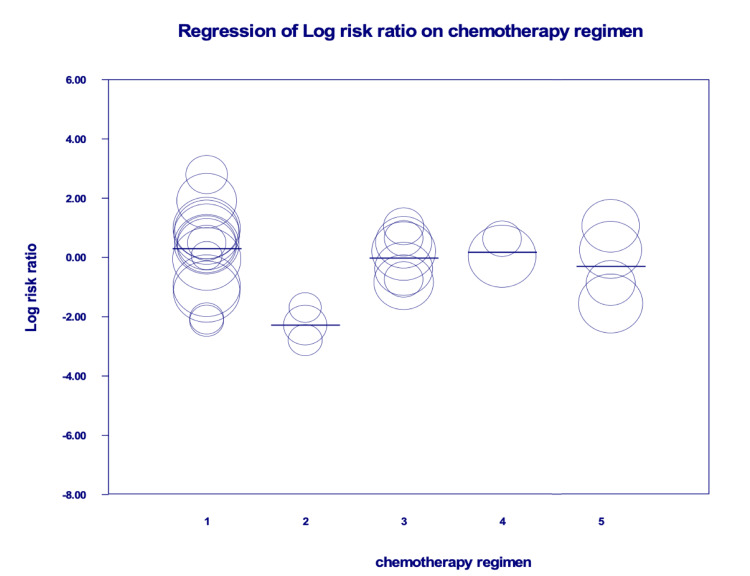
Meta-regression of chemotherapy regimens. Regimen 1: 5-FU, LV plus oxaliplatin; Regimen 2: intra-arterial 5-FU and oxaliplatin; Regimen 3: CPT-11, 5-FU, and LV; Regimen 4: venous 5-FU and arterial FUDR; Regimen 5: 5-FU, I-OHP, and LV. 5-FU: fluorouracil; LV: leucovorin (folic acid); CPT-11: irinotecan; FUDR: floxuridine; I-OHP: oxalatoplatinum

The overall risk of grade 3 and 4 toxicities was not different between both groups (RR = 1.00, 95% CI = 0.57-1.75). Meta-regression (Table 10, Figure 10) showed that Tegimen 2 (i.e. intra-arterial 5-FU and oxaliplatin) had significantly less risk of toxicity (p = 0.0048). Of note, one study reported the incidence of grade 3 or 4 main toxicities was greater by 15.3% (95% CI = 7.5-23.2) in women compared to men from the chronomodulated treatment group [44].

**Table 10:** Meta-regression of chemotherapy regimens. Control chemotherapy regimen: 5-FU, LV plus oxaliplatin; chemotherapy regimen 2: intra-arterial 5-FU and oxaliplatin; chemotherapy regimen 3: CPT-11, 5-FU, and LV; chemotherapy regimen 4: venous 5-FU and arterial FUDR; chemotherapy regimen 5: 5-FU, I-OHP, and LV. 5-FU: fluorouracil; LV: leucovorin (folic acid); CPT-11: irinotecan; FUDR: floxuridine; I-OHP: oxalatoplatinum

Covariate reference	Coefficient	Standard error	95% Lower	95% Upper	Z-value	Two-sided p-value	Set
Intercept	0.2952	0.2579	-0.2102	0.8007	1.14	0.2522	
Chemotherapy regimen: 2	-2.5785	0.9144	-4.3706	-0.7864	-2.82	0.0048	Q = 10.45, df = 5, p = 0.0635
Chemotherapy regimen: 3	-0.3154	0.4858	-1.2675	0.6367	-0.65	0.5162	Q = 10.45, df = 5, p = 0.0635
Chemotherapy regimen: 4	-0.1188	0.7673	-1.6227	1.3851	-0.15	0.877	Q = 10.45, df = 5, p = 0.0635
Chemotherapy regimen: 5	-0.5965	0.547	-1.6686	0.4757	-1.09	0.2755	Q = 10.45, df = 5, p = 0.0635

Discussion

In this meta-analysis of seven RCTs, there was no significant advantage of chronomodulated chemotherapy in improving the response rate and gastrointestinal, neurological, and skin toxicities. However, haematological toxicity was significantly lower compared to the conventional regimen.

Liao et al. assessed the overall survival and safety of patients on chronomodulated chemotherapy who were more prone to diarrhoea but at less risk of neutropenia. There was no difference in overall survival and response rate [6]. In contrast with the Liao et al. study in which IV administration was the only method of delivery, this review included two extra RCTs comparing chronomodulated and conventional intra-arterial administration of chemotherapeutic agents. Moreover, the effect of different chemotherapeutic regimens on toxicity was studied in meta-regression which suggested better tolerance of intrahepatic artery versus systemic delivery of 5-FU.

There were several advantages for patients on the chronomodulated regimen. They were three times less likely to develop neutropenia and developed less stomatitis compared to the conventional regimen. Furthermore, the number of withdrawals was significantly higher in the non-chronomodulated group due to either severe toxicity or disease progression [29,44].

Giacchetti et al. included the largest number of patients in their trial (564 patients) and reported a significantly (15.3%, 95% CI = 7.5-23.2) greater incidence of grade 3 and 4 toxicity incidence in women [44]. Moreover, the overall mortality was higher in women than in men (38% vs. 25%, p < 0.01) and the same effect was noticed in progression-free survival in the chronomodulated arm [44]. This may suggest a strong role of gender in the efficacy of chronomodulation and paves the way for future studies.

Intrahepatic arterial administration of chemotherapy for colorectal liver metastasis was reported to be more effective than systematic administration [47]. The meta-regression found a significantly lower risk of toxicity in patients who were administered intra-arterial 5-FU and oxaliplatin. However, this was not the case for patients who were administered intra-arterial FUDR and venous 5 FU, suggesting more tolerability for 5-FU if given intra-arterial rather than systemic. The influence of previous chemotherapy on the effect of chronomodulation is not clear. Focan et al. reported a significantly larger number of patients with previous chemotherapy in the chronomodulation group. This may have masked the difference in ORR and toxicity reported in other trials in which they matched the distribution of the number of patients who had previous chemotherapy among both groups [45].

The activity of many enzymes regulating the anabolism and catabolism of agents such as 5-FU and oxaliplatin has shown a circadian variation [11,32]. At least 50% of the proportion of cells in the S-phase change during the day [48]. Therefore, circadian rhythms can alter the tolerability of patients to chemotherapy and improve its anti-tumour efficacy when administrated near their respective times of best tolerability [25]. The time of best tolerability and efficacy depends on the circadian changes of enzymes involved in the metabolism of each agent [7,49]. This was the predominant reason behind the scheduled chronomodulated chemotherapy at different peak doses considering the chemotherapeutic agent (e.g. peak dose at 04:00 for 5-FU and at 16:00 for oxaliplatin) [5,29].

This review has some limitations. According to the risk of bias assessment (Table 6), there were some concerns in the majority of studies and a high concern of bias in one study. There was also considerable heterogeneity in the meta-analysis which is not explained by the meta-regression for different chemotherapeutic agents as the R^2^ value was 0 (Table 10); therefore, the random effects model was used. Potential confounding factors such as previous chemotherapy and the volume and location of distant metastasis were not reported in all studies. All included RCTs calculated their sample size based on ORR as a primary outcome which may not be sufficient to study toxicity. Further studies are needed to address the effect of gender and other disease-specific factors on chronomodulation. Age and disease stage-specific characteristics should be taken into consideration to explore their impact on the usefulness of this approach to improve the outcome for patients who will benefit from chronomodulated chemotherapy with advanced colorectal cancer. In addition, comparisons of some secondary outcomes comparisons, such as neutropenia were based only on a limited number of studies. Despite meta-regression conducted to examine the effect of different chemotherapeutic regimens, this may be influenced by the underlying heterogeneity of the disease stage. Hence, future studies need to consider disease stages among other disease and patient characteristics. This consideration may allow a better assessment of the influence of the different chemotherapeutic regimens and their delivery approach.

## Conclusions

There was no difference in ORR and overall toxicity between chronomodulated and non-chronomodulated chemotherapy used in patients with advanced colorectal cancer. Chronomodulated chemotherapy can be considered in patients at high risk of developing haematological toxicities. Chronomodulation may be more tolerable in men. Further high-quality studies are recommended to confirm the current findings.
